# Prevalence and Risk by Age and Sex of Sleep Dysregulation and Depressive Episodes in Bipolar and Depressive Disorders in a Community Survey in Sardinia, Italy

**DOI:** 10.3390/jcm13164870

**Published:** 2024-08-18

**Authors:** Patrizia Congiu, Mauro Giovanni Carta, Alessandra Perra, Elisa Cantone, Stefano Lorrai, Elisa Pintus, Massimo Tusconi, Giulia Cossu, Stefania Redolfi, Federica Sancassiani

**Affiliations:** 1 Department of Medical Sciences and Public Health, Sleep Disorder Research Center, University of Cagliari, 09124 Cagliari, Italy; patcongiu@gmail.com (P.C.); stefania.redolfi@unica.it (S.R.); 2 Sleep Outpatient Service, Clinica Tommasini di Jerzu, 08044 Jerzu, Italy; 3 Department of Medical Sciences and Public Health, Section of Psychiatry, University of Cagliari, 09124 Cagliari, Italy; maurogcarta@gmail.com (M.G.C.); alessandra.perra@unica.it (A.P.); elisa.cantone@libero.it (E.C.); stefanolorrai@live.it (S.L.); pintuselisa@yahoo.it (E.P.); giuliaci@hotmail.com (G.C.); federicasancassiani@yahoo.it (F.S.); 4 University Hospital of Cagliari, 09042 Cagliari, Italy

**Keywords:** mood disorders, insomnia, PHQ9, patient health questionnaire

## Abstract

**Background/Objectives**: Sleep disturbances often accompany mood disorders and persistent insomnia after mood symptoms have resolved may be a marker of poor outcome. The association between sleep symptoms and mood disorders seems to change with age and sex. This study aims to assess the frequency of depressive episodes and sleep disorders in the general population through an agile screening questionnaire and to evaluate the association of depressive episodes and sleep symptoms by sex and age categories. **Methods**: 774 women and 728 men from Sardinia aged > 16 years old were enrolled. The Patient Health Questionnaire (PHQ-9) was administered through a computer-assisted telephonic interview. **Results**: The frequency of depressive episodes was double in women (10.6% vs. 4.4%; *p* < 0.0001), with the highest values in women > 75 yo (17.4%). The frequency of sleep dysregulation was double in women (18.7% vs. 9.6%; *p* < 0.0001), with the highest values in women > 75 yo (35.9%) and the lowest in the group of men > 75 yo. The group of young males showed the lowest frequency of depressive episodes (1.4%) and a frequency of sleep dysregulation (9.1%) similar to that of the other groups of age and sex. Sleep dysregulation without depressive episodes presented a higher distribution in the elderly, both in males (20.7%) and in females (18.5%). No significative differences were found across sex and age groups in the distribution of depressive episodes without sleep dysregulation. **Conclusions**: The use of an agile screener such as PHQ9 in the general population and/or in populations at risk can be a valuable tool in finding those individuals in whom sleep dysregulation may represent an early warning signal, one that may be thoroughly evaluated to identify and treat possible sleep disorders early.

## 1. Introduction

The triumph of the descriptive approach in psychiatry has made it possible to develop diagnostic shared systems allowing for scientific communication; it has anchored therapeutic and rehabilitative practices and the evaluation of the effectiveness of treatments to a medical model, although this has not been without criticism [1,2]. The descriptive definition of a syndrome does not always align with the medical concept of disease, which requires both etiopathological understanding and anatomical or functional description. Historically, this led to the inclusion of unrelated conditions under a single diagnosis, like jaundice. However, despite these challenges, a descriptive approach in mental health has enabled treatment validation and facilitated epidemiological studies to assess public health impacts and associated risk factors.

However, descriptive diagnoses are heuristic categories in terms of the hypotheses of the underlying diseases. In epidemiology, it is useful to compare the results of investigations conducted with descriptive categorical diagnoses with those conducted based on more open syndromic descriptions [3,4]. If this is true in general, it has an even more significant meaning when talking about depressive disorders and the relationship between depressive disorders and insomnia.

There is a broad debate about the classification of depressive and mood disorders. [5,6] The classification of the American Psychiatric Association, DSM-5 in the last version [7] marked a net distinction between bipolar and depressive disorders; against this perspective, the so-called neo-Kraepelinian approach includes in the “spectrum of bipolar disorders” most depressive disorders and ADHD [8,9,10] but also sees the spectrum as a continuum between mood disorders, subthreshold pictures and hyperthymic nonpathological temperaments [11,12,13]. In this context, DYMERS, a stress-related syndrome marked by hyperactivity and disrupted biological rhythms, has been identified as an intermediate state between normal stress and pathology, particularly linked to bipolar spectrum disorders [12,14,15]. Major depressive episodes, characterized by at least five significant depressive symptoms persisting for at least 15 days, can episodically occur in both major depressive disorder and bipolar disorder. However, a similar syndrome can also be found in stress-related adjustment disorder [16,17,18]. Furthermore, it is recognized that lifetime diagnoses (such as those of bipolar disorder or major depressive disorder) lead to the problem of recall bias in epidemiological studies [19,20]. Although epidemiological studies offer estimates on the global prevalence of mood disorders based on descriptive approaches, the underlying foundations are not always robust. Nonetheless, using simple screening tools to estimate the frequency of depressive episodes can still provide valuable heuristic insights [21].

Major depressive disorder, as well as bipolar disorders, is frequently accompanied by sleep symptoms such as insomnia or hypersomnia/excessive daytime sleepiness. Sleep disorders were found to be associated with depressive disorders, with a hazard ratio for healthy people against major depressive disorders of 0.34 (CI95% 0.31–0.37) [22] and for insomnia symptoms in people with depressive disorders of 1.437 [CI95% 1.064–1.940] [23], but the extent of this association is not confirmed by several surveys; additionally, knowledge of the underlying mechanism as well the role of multiple lifestyle factors in it remain unclear [22]. In many people, insomnia persists even after mood symptoms have resolved and it may be a marker of poorer outcomes [24,25] than in people with previous mood disorders without the persistence of sleep disorders [26,27].

Sleep disorders include conditions in which the quality, quantity and duration of sleep during the night are compromised with diurnal consequences. According to a descriptive-pathophysiological approach, the International Classification of Sleep Disorders ICSD-3-TR [28,29] classify sleep disorders in six main categories:

- Insomnia: difficulty in falling or staying asleep despite adequate opportunities, leading to daytime impairment.
- Sleep-related breathing disorders: abnormal respiration during sleep, detected by polysomnography (PSG).
- Central disorders of hypersomnolence: excessive daytime sleepiness not due to other sleep or circadian rhythm disorders, causing difficulties in staying alert.
- Circadian rhythm sleep–wake disorders: misalignment between the internal circadian rhythm and required sleep timing, causing difficulties in falling asleep or waking up on time.
- Parasomnias: physical actions or verbal expressions during sleep, such as talking, walking, or eating.
- Sleep-related movement disorders: movements that disrupt the ability to fall or stay asleep.

A systematic review found the prevalence of sleep disorders that occurred three days per week to be the following: difficulty falling asleep 14.0%, difficulty staying asleep 28.3%, waking up too early, 32.1% and non-restorative sleep, 39.9%. If restrictive criteria by time were included, the prevalence dropped to 8.4% for non-restorative sleep and to 12.9% for difficulty falling asleep. The prevalence of the disorders was 22.1% according to DSM-IV-TR [30], 4.7% according to ICD-10 and 15.1% according to ICSD-2; approximately 22% had a sleep disorder according to one of the three classification criteria, while the prevalence of insomnia disorder according to the DSM-5 was 10.8% [31].

Considering that depressive episodes are common across various disorders, such as major depression and bipolar disorder, and are subject to recall bias in epidemiological studies, a descriptive transdiagnostic approach may be useful for studying the frequency and association between depressive episodes and sleep disorders. Furthermore, the association between sleep disorders and mood disorders seems to change with age [32] and sex [23] or, according to another perspective, the extent of the symptomatologic component linked to sleep in mood disorders seems to change with age and sex [26]. Studying the here and now of the association between sleep disturbances and current depressive episodes by sex and age in a community survey can offer many insights [33,34,35]. The prospect of a community survey may also allow us to clarify aspects of the relationship between current depression and depressive symptoms of depression [36].

It is important in this context to add that assessment and screening of depression with scales are appropriate and correct, but their reliability is to be recognized in non-universal settings and to be evaluated in the clinical setting in each individual case [37].

The aim of this study is to measure the frequency of depressive episodes and sleep disorders through a screening questionnaire in a general population sample and to study the association of depressive episodes by sex and age categories.

## 2. Materials and Methods

Sample: The community survey was conducted by phone in Sardinia, Italy. The study involved people aged 16 and older living in households with telephone-wire connections (at that time, a still frequent method). Sampling was achieved with a strata technique. The population of the whole region was broken down into 16 geographically defined strata representing the capital city of the eight official provinces plus the villages of each province. The number of respondents recruited from each stratum corresponded to the proportion of the population living in the capital of the province or villages of that province, according to the Italian National Institute of Statistics [38]. Each stratum was subdivided into ten sex and five age group cells (16–29, 30–44, 45–59, 60–74, >75 by male and female). A random sample was drawn from all registered private household telephones. As soon as a cell was saturated, persons who did not meet age and sex criteria were excluded from the database of those eligible for interview (N = 2343), selected on the basis of regional registries. We obtained due to the particularly balanced nature of the study a high percentage of participants (N = 1502) from the initial sample (N = 2343), differentiating the two initial populations based on their consent to participation.

The interviewers were medical doctors, psychologists and post-graduate students in medicine.

Ethics: Participants had to sign informed consent. This study was evaluated and approved by the Ethics Committee of the University Hospital of Cagliari. This research was conducted according to the Helsinki Declaration.

Interview: Computer-assisted telephone interviewing (CATI) was used. The main objective of the protocol was the perception of depression in the general population; for this purpose, an instrument was adopted with case vignettes, which were carefully described in the previous works produced by this research [39,40].

The presence of depressive symptoms during the past two weeks was measured using the Patient Health Questionnaire (PHQ-9) [41], Italian version [42,43]. Respondents indicate that for each of the nine depressive symptoms (corresponding to the core criteria of DSM classification), the degree of presence of the symptom on a Likert scale is from 0 (not at all) to 4 (nearly every day). A cut-off of 9/10 identifies people with a high likelihood of having a depressive episode of at least moderate level [41]. Item 3 of the PHQ9 measures the issues of sleep (“Trouble falling or staying asleep or sleeping too much)”.

Statistical Analysis: The frequency of the response to item 3 (indicative of the average frequency of the score on the sleep disorders item) as well as the total score on PHQ9 (indicative of the average frequency of the score relating to general symptoms) was compared in the different groups by age and sex were compared using 1-way ANOVA. For this purpose, the age group over 75 was used as a pivot for both sexes.

The frequency of people who scored more than nine on the PHQ9 (score indicative of a medium to severe depressive episode) was compared between the age and sex groups. Also, in this case, the >75 age class was used as a pivot for both sexes, but a comparison was also conducted between the classes most divergent in frequency. Statistical analysis was conducted with chi-square tests using Yates’ continuous correction where required.

The frequency of people who scored more than one at Item 3 of the PHQ9 (indicative of having “trouble falling or staying asleep or sleeping” from more than half the days [coded 2] to nearly every day [coded 3]) was compared between the age and sex groups. Also, in this case, the >75 age class was used as a pivot for both sexes, but a comparison was also conducted between the classes most divergent in frequency. Statistical analysis was conducted with chi-square tests using Yates’ continuous correction. The frequency of Men Depressive Episodes without Sleep Disturbance (<2 item 3 PHQ) as well as the frequency of Sleep Disturbance Without Depressive Episodes was also compared with a similar methodology between groups by sex and age. In this case, given the small number of some subgroups, in addition to the chi-square with possible Yates correction, Fisher’s exact test was necessary in some cases.

## 3. Results

Table 1 describes the sample interviewed based on distribution by sex and age. It should be noted that those over sixty represent around 20% of the male sample and 30% of the female sample. On the other hand, young people (16–29 years) do not reach 20% in both sexes.

Table 2 shows the distribution of the overall score of the PHQ9 * by age and sex.

In males, the oldest category (>75) shows a tendency towards a higher frequency than the other categories, but only among the youngest (16–19) does the difference reach the statistical significance (3.58 ± 3.56 vs. 2.66 ± 2.42, *p* = 0.036). In women, older women (>75) present a higher frequency in all categories except for “old youngers” (60–74), i.e., >75 vs. 45–59 (5.41 ± 4.83 vs. 4.66 ± 4.44, *p* = 0.003); >75 vs. 30–34 (5.41 ± 4.83 vs. 4.16 ± 3.86, *p* = 0.017); >75 vs. 16–29 (5.41 ± 4.83 vs. 4.03 ± 3.72, *p* = 0.015). Comparing the difference by sex in the same age categories, a greater frequency was found in women except among the youngest older adults: (>75) women 5.41 ± 4.83 vs. men 3.58 ± 3.56 (*p* = 0.014); (60–74) women 4.66 ± 4.44 vs. men 2.87 ± 3.02 (*p* < 0.0001); (30–44) women 4.16 ± 3.86 vs. men 2.87 ± 3.44 (*p* = 0.002); (16–29) women 4.03 ± 3.72 vs. men 2.66 ± 2.42 (*p* < 0.001). When the mean score of item 3 of PHQ9 by age and sex is analyzed in men, a distribution not ideally like the previous one is highlighted (see Table 3).

In males, people over 75 years old show a higher frequency than other age groups, but the difference is statistically significant only compared to people between 30 and 44 years old (0.68 ± 0.67 vs. 0.42 ± 0.73, *p* = 0.015). In women, the older ones show a very high average score (1.19 ± 10.2); the difference is statistically significant compared to all the other age categories, i.e., 60–74 (0.71 ± 0.88, *p* < 0.001); 45–59 (0.81 ± 1.02; *p* = 0.002); 30–44 (0.63 ± 0.83; *p* < 0.001); 16–29 (0.59 ± 0.91; *p* < 0.001).

The distribution of depressive episodes by sex and age (PHQ9 > 9), as shown in Table 4, is not homogeneous with the mean distribution of the PHQ total score presented in Table 2.

In males, the frequency in the older age group (3.4%) is homogeneous with that of the other age groups. In contrast, the highest frequency was found in the young elderly aged 60–74, which shows a statistically significant difference towards those younger of 16–29 years (6.1% vs. 1.4%, *p* = 0.040). In women, on the contrary, the older ones (>75) show a very high frequency, but this reaches a statistically significant difference only compared to young people aged 16–19 (17.4% vs. 6.6%, *p* = 0.006). Compared to males, women present a higher frequency of depressive episodes in all age groups except among younger elderly people (60–74 years), as illustrated in Table 4. This finding is in agreement with data in the literature; several epidemiological studies conducted through structured clinical interviews corroborate the finding [44,45]. In the total sample, the frequency of depressive episodes in females is double that of males.

In males, the distribution of sleep dysregulation (PHQ Item 3 < 2) by age and sex (Table 5) presents some aspects similar to the distribution of depressive episodes with older people (>75) who do not present higher frequencies than other groups of age. The young elderly (60–74) have the highest peak; however, this peak only reaches a statistically significant difference compared to the 30–44 group (14.3% vs. 7.6%, *p* = 0.043) and not in comparison with the youngest (16–19) (14.3% vs. 9.1%, chi-square with Yates correction 1.348; *p* = 0.246; OR = 1.67; CI95% 0.8–3.5).

In women, according to the distribution of depressive episodes, sleep dysregulation has the highest frequency peak among the oldest (35.9%), but in this case, a statistically significant difference is achieved compared to all other age groups. However, the distribution in the other age groups is not homogeneous with women 45–59 years old with a prevalence (24.2%) higher against the 30–44-year-old group (12.6%, *p* = 0.008) than in the younger 16–29-year-olds (prevalence 12.6%, *p* = 0.008). In the comparison by sex, women present a higher frequency only in the groups >75 (35.9% vs. 6.9%; *p* < 0.0001) and 45–59 (24.2% vs. 9.7%; *p*< 0.0001). In the total sample, however, women were twice as frequent as men.

Table 6 shows that no differences are found across sex and age groups in the distribution of depressive episodes without sleep dysregulation (i.e., people with PHQ > 9 but Item3 score < 2). Sleep dysregulation without depressive episodes presents a higher distribution in the elderly, both in males (20.7%) and in women (18.5%) with statistically significant differences compared to all other groups but without evidence of a progressive prevalence by age instead of a homogeneous distribution under 75 years. There is no statistically significant difference by sex in the total sample; the frequency is higher amongst males (20.7% vs. 8.5%) but at the limits of statistical significance (*p* = 0.097).

## 4. Discussion

This study reports that current depressive episodes are about 10% in females and 5% in males, with sleep dysregulation occurring at twice the rate, showing a similar female/male ratio. These prevalence rates and demographic distributions align closely not only with recent community surveys using similar methods in Spain [46], a nation with a cultural and economic background not far from Sardinia, but also are even quite similar to those conducted in countries less close on a cultural and economic level such as Brazil [47] or Russia [48]. Surprisingly, the few community surveys conducted with screening tools show less variability in results than the range of point prevalence of depressive episodes detected with structured clinical interviews [49]. However, a study recently analyzed the comparability of risk profiles in people who tested positive for PHQ9 compared to people identified as having a depressive episode through a structured interview [50]. The results show that the identified cases of depressive episodes by PHQ-9 were not reliable with CIDI-SF [51] identified Major Depressive Disorder cases. Even the two sets of symptoms identified with the different tools had different factor structures in genomic modeling. Major depressive symptoms found using the PHQ-9 were more homogeneous, describing general dysphoria, while CIDI SF symptoms were more informative on within-major depression heterogeneity [50]. The link between PHQ-9 positivity and the dysphoric component of mood disorders is crucial for studying sleep dysregulation. Dysphoria and disruptions in individual rhythms, including sleep, are key elements in the borderline area between pathology and normalcy, which is central to the bipolar spectrum according to a neo-Kraepelinian approach [52,53,54,55]. However, the same elements are also relevant in the psychopathology of that syndrome defined by hyperactivity and dysregulation of rhythms (DYMERS), which is today seen as a border between pathology and well-being but in a broader vision of the previous concept of bipolar spectrum as a possible prodromal condition not only of the disorder bipolar [15,56,57]. The similarity in age and gender distribution between depressive episodes identified by PHQ-9 and sleep dysregulation from the same instrument suggests, if not proves, that the screening test primarily detects dysphoric depression, which is closer to the bipolar spectrum [58,59,60], while the second is an element closely linked to this area which can in many cases be an antecedent of the frank pathology it is known that dysregulation of rhythms can be a factor triggering crises [61] or even linked to the onset of bipolar disorders [62].

The distribution of depressive disorders, depressive symptoms and sleep dysregulation by sex and age shows similar trends across groups, except for young males, who have a lower frequency of depressive episodes but similar rates of sleep dysregulation compared to other age groups. This discrepancy suggests that sleep dysregulation may be a risk factor for mood disorders, leading to higher sleep issues and fewer mood disorders in younger individuals. Further investigation is needed to understand why this pattern is more pronounced in younger males and why the ratio does not change progressively with age. It must be underlined that the use of substances, in particular stimulants such as cocaine, begins earlier in males with a ratio of up to 4/1 compared to females in adolescence [63,64], as well as unhealthy behaviors that can potentially expose one to sleep dysregulation such as compulsive internet use and video game playing; additionally, smoking, cannabis use and being bullied were found to be more frequent among males [65]. However, these behaviors, including the excessive use of video games and smartphones and the shift in the sleep–wake cycle with exposure to light pollution, are still present in both sexes in the young generations of the current era [66].

Suppose we observe the trend in the prevalence of sleep disorders without episodes of depression in both sexes. In that case, we note a higher frequency in both sexes in the young ages 16–29 (8% in women, 8.4% in males), followed by a decrease in the 30–44 age group (5.8% in women, 4.7% in males), followed by a peak in 45–59 but only in women (14.7% women, 6.5% males), followed by a leveling out of the frequency in young older adults 60–74 (8% women, 9.8% men) and then a rise to the maximum levels in older >75 (18.5% and 20.7% respectively). Hyperactivation around the use of substances [67,68,69], the subversion of the circadian rhythm and the use of smartphones, both factors of light pollution, are probably the cause of the high prevalence [70,71,72] in young people; peri-menstrual hormonal factors are probably related to the peak in women [73]. It is more difficult to explain the high frequency of sleep disorders in old age (>75), which, moreover, does not correspond to a prevalence of depressive episodes in the same age groups. The factors more likely to contribute to increasing the prevalence of sleep complaints in older adults are (1) the higher prevalence of comorbidities [74] and associated drugs that may negatively impact nocturnal sleep or that may interfere with daytime activities, thus causing an alteration/disruption of the 24 h sleep–wake pattern; (2) retirement, which, with its typical changing in lifestyle, may lead to inadequate sleep hygiene and consequently to poor sleep; (3) changes in sleep architecture in older adults with sleep fragmentation, increased arousals, decreased slow-wave sleep and reduced sleep efficiency [75]; (4) alterations in circadian rhythms with advancing of the leading sleep episode and consequent earlier evening sleepiness and morning awaking [76]. The higher prevalence of sleep disorders in women >75 years old may be partly attributed to the better-preserved quality of NREM sleep in older men [77,78]; it was reported in a recent meta-analysis that insomnia symptoms were most frequent in people >65 years old, especially in women.

While the utility and necessity of using depression scales for screening and assessing patients are evident, this approach should not be applied universally. Instead, it must be evaluated on a case-by-case basis, tailored to the specific clinical and diagnostic needs of each individual [79,79,80].

This study is subject to several limitations. First, the telephone-based nature of the community survey may have introduced selection bias, as individuals without access to telecommunication services or those unwilling to participate in telephone interviews might have been underrepresented. This limitation could affect the generalizability of the findings to the broader population. Additionally, the self-administered PHQ-9 scale, while widely used for assessing depressive symptoms, relies on participants’ self-reports, which may be influenced by response biases, such as social desirability or varying levels of self-awareness.

It is known that in the depression of older people, somatic symptoms dominate to the detriment of affective symptoms [81] and a careful analysis of the results of an extensive epidemiological survey conducted in the USA led the authors to state that the low estimated prevalence of major depressive episodes among the elderly is due to an increase in confounding with physical disorders [82]. From this point of view, sleep disorders, even in the absence of a diagnosis of depression, could be a helpful signal in primary prevention in young people or an indicator of a need for greater diagnostic attention in the elderly, also given the high incidence in this age of dramatic consequences of untreated and unrecognized depressive episodes.

## 5. Conclusions

The use of an agile screener such as PHQ9 in the general population and/or in populations at risk can be a valuable tool in finding those individuals in whom sleep dysregulation may represent an early warning signal and that may be thoroughly evaluated to identify and treat possible sleep disorders early. These disorders represent an important trigger of depressive symptoms, as well as an important factor of aggravation of the symptomatology and are an important clinical element to be identified and treated at an early stage in order to improve the general clinical picture and obtain an optimal outcome. Future prospective studies will need to confirm the suggestions emerging from this investigation.

## Figures and Tables

**Table 1:** Characteristics of the sample by age and sex.

Age	Men	Women
>75	58 (8.0%)	92 (11.9%)
60–74	132 (18.1%)	150 (20.2%)
45–59	185 (25.4%)	190 (25.5%)
30–44	211 (30.0%)	206 (27.7%)
16–29	142 (19.5%)	136 (17.6%)
Total	728	774

**Table 2:** Distribution of the overall score of the PHQ9* by age and sex.

Age	Men	Total PHQ9 Men	ANOVA 1way within Men by Age Classes	Women	Total PHQ9 Women	ANOVA 1way within Women by Age Classes	Men vs. Women
>75	58 (8.0%)	3.58 ± 3.56	Pivot	92 (11.9%)	5.41 ± 4.83	Pivot	df 1148 F = 6.197 *p* = 0.014
60–74	132 (18.1%)	2.93 ± 3.52	df 1188 F = 1.365 *p* = 0.244	150 (20.2%)	3.71 ± 3.85	df 1240 F = 9.239 *p* = 0.003	df 1280 F = 3.122 *p* = 0.078
45–59	185 (25.4%)	2.87 ± 3.02	df 1241 F = 2.235 *p* = 0.136	190 (25.5%)	4.66 ± 4.44	df 1280 F = 1.714 *p* = 0.192	df 1373 F = 20.730 *p* < 0.0001
30–44	211 (30.0%)	2.87 ± 3.44	df 1267 F = 1.909 *p* = 0.168	206 (27.7%)	4.16 ± 3.86	df 1296 F = 5.773 *p* = 0.017	df 1415 F = 9.825 *p* = 0.002
16–29	142 (19.5%)	2.66 ± 2.42	df 1198 F = 4.458 *p* = 0.036	136 (17.6%)	4.03 ± 3.72	df 1226 F = 6.004 *p* = 0.015	df 1276 F = 13.358 *p* < 0.0001

* PHQ = Patient Health Questionnaire.

**Table 3:** Distribution of the means score of item on dysregulation of sleep (PHQ9 * Item 3) by age and sex.

Age	Men	Item 3 PHQ9 Men	ANOVA 1way within Men by Age Classes	Percentage of Total PHQ9 Score	Women	Item 3 PHQ9 Women	ANOVA 1way within Women by Age Classes	Percentage of Total PHQ9 Score	Men vs. Women
>75	58 (8.0%)	0.68 ± 0.67	Pivot	19.0%	92 (11.9%)	1.19 ± 1.02	Pivot	22%	df 1, 148 F = 11.387 *p* = 0.001
60–74	132 (18.1%)	0.67 ± 0.91	df 1188 F = 0.006 *p* = 0.940	22.8%	150 (20.2%)	0.71 ± 0.88	df 1240 F = 15.011 *p* < 0.0001	19.1%	df 1, 280 F = 0.141 *p* = 0.708
45–59	185 (25.4%)	0.50 ± 0.77	df 1241 F = 2.560 *p* = 0.111	17.4%	190 (25.5%)	0.81 ± 1.02	df 1338 F = 9.314 *p* = 0.002	17.4%	df 1, 373 F = 10.990 *p* = 0.001
30–44	211 (30.0%)	0.42 ± 0.73	df 1267 F = 5.972 *p* = 0.015	14.6%	206 (27.7%)	0.63 ± 0.83	df 1301 F = 25.267 *p* < 0.0001	15.1%	df 1, 415 F = 7.536 *p* = 0.006
16–29	142 (19.5%)	0.52 ± 0.81	df 1198 F = 1.767 *p* = 0.185	19.5%	136 (17.6%)	0.59 ± 0.91	df 1226 F = 21.625 *p* < 0.0001	14.6%	df 1, 280 F = 0.141 *p* = 0.708

* PHQ = Patient Health Questionnaire.

**Table 4:** Distribution of depressive episodes (PHQ9 > 9) * by age and sex.

Age	Men	Men Depressive Episodes (PHQ > 9)	Within Men by Age Classes, Chi-square with Yates Correction if Needed.	Women	Women Depressive Episodes (PHQ > 9)	Within Women by Age Classes, Chi-Square with Yates Correction if Needed.	Women vs. Men (Chi-Square with Yates Correction if Needed)
>75	58 (8.0%)	3.4% (2/58)	Pivot	92 (11.9%)	17.4% (16/92)	Pivot	
60–74	132 (18.1%)	6.1% (8/132)	0.152, *p* = 0.697 OR = 0.55 (CI95% 0.1–2.7)	150 (20.2%)	9.3% (14/150)	3.409, *p* = 0.105 OR = 2.04 (CI95% 0.9–4.4)	1.997, *p* = 0.158 OR = 1.77 (CI95% 0.8–3.9)
45–59	185 (25.4%)	4.3% (8/185)	0.086, *p* = 0.769 OR = 0.79 (CI95% 0.2–3.8)	190 (25.5%)	10.5% (20/190)	2.653, *p* = 0.065 OR = 1.78 (CI95% 0.9–3.6)	6.549, *p* = 0.010 OR = 5.89 (CI95% 1.3–26.6)
30–44	211 (30.0%)	5.7% (12/211)	0.462, *p* = 0.497 OR = 0.59 (CI95% 0.2–3.8)	206 (27.7%)	11.1% (23/206)	2.168, *p* = 0.141 OR = 1.67 (CI95% 0.8–3.7)	4.068, *p* = 0.044 OR = 2.08 (CI95% 1.0–4.3)
16–29	142 (19.5%)	1.4% (2/142)	0.143, *p* = 0.705 OR = 2.50 (CI95% 0.3–18.1)	136 (17.6%)	6.6% (9/136)	7.462, *p* = 0.006 OR = 3.18 (CI95% 1.3–7.5)	4.960, *p* = 0.026 OR = 4.96 (CI95% 1.0–4.3)
Total		4.4% (32/728)			10.6% (82/774)		20.052, *p* < 0.0001 OR = 2.57 (CI95% 1.7–3.9)
High divergences		60–74 vs. 16–29	4.210, *p* = 0.040 OR = 4.5 (CI95% 1.0–21.7)		30–44 vs. 16–29	1.997, *p* = 0.141 OR = 1.77 (CI95% 0.8–3.9)	

* PHQ = Patient Health Questionnaire.

**Table 5:** Distribution of sleep dysregulation (PHQ9 Item 3 < 2) * by age and sex.

Age	Men	Men Sleep Disorders (PHQ Item 3 > 1)	Within Men by Age Classes, Chi-Square with Yates Correction if Needed.	Women Sleep Disorders (PHQ Item 3 > 1)	Within Women by Age Classes, Chi-square with Yates Correction if Needed.	Women vs. Men (Chi-Square with Yates Correction if Needed)
>75	58 (8.0%)	6.9% (4/58)	Pivot	35.9% (33/92)	Pivot	16.070, *p* < 0.0001 OR = 7.55 (CI95% 2.5–22.7)
60–74	132 (18.1%)	14.3% (19/132)	2.656; *p* = 0.103 OR = 0.40 (CI95% 0.1–1.2)	14.7% (22/150)	14.598, *p* < 0.0001 OR = 3.24 (CI95% 1.7–6.1)	0.004; *p* = 0.940 OR = 1.02 (CI95% 0.5–2.0)
45–59	185 (25.4%)	9.7% (18/185)	0.430; *p* = 0.512 OR = 0.69 (CI95% 0.2–2.1)	24.2% (46/190)	4.178, *p* = 0.041 OR = 1.75 (CI95% 1.1–3.0)	13.887, *p* < 0.0001 OR = 2.96 (CI95% 1.6–5.3)
30–44	211 (30.0%)	7.6% (16/211)	2.064; *p* = 0.151 OR = 0.44 (CI95% 0.1–1.4)	12.6% (26/206)	21.647, *p* < 0.0001 OR = 3.87 (CI95% 2.1–7.0)	2.921; *p* = 0.087 OR = 1.95 (CI95% 0.9–3.4)
16–29	142 (19.5%)	9.1% (13/142)	0.270; *p* = 0.603 OR = 0.73 (CI95% 0.2–2.4)	12.6%, (17/136)	15.065, *p* < 0.0001 OR = 3.58 (CI95% 1.8–7.0)	0.807; *p* = 0.369 OR = 1.42 (CI95% 0.7–3.0)
Total		9.6% (70/728)		18.7% (144/774)		24.812, *p* < 0.0001 OR = 2.15 (CI95% 1.6–2.9)
High divergences		60–74 vs. 30–44	4.111; *p* = 0.043 OR = 0.2.05 (CI95% 1.0–4.1)	45–59 vs. 16–29° and 30–44°	6.972, *p* = 0.008 OR = 2.23 (CI95% 1.2–4.9) 8.924. *p* = 0.008 OR = 2.21 (CL95% 1.3–3.7)	

* PHQ = Patient Health Questionnaire.

**Table 6:** Distribution by age and sex of depressive episodes without sleep dysregulation (people with PHQ > 9 *** and Item3 < 2) and of sleep dysregulation (PHQ9 Item 3 > 1) without depressive episodes (PHQ9 < 10).

Age	Men	Men’s Depressive Episodes without Sleep Disturbance (PHQ It. 3 < 2)	Men by Age (Fisher Exact Test) p	Men Sleep Disturbance Without Depressive Episode	Men by Age (Fisher Exact Test) p	Women	Women Depressive Episodes without Sleep Disturbance (PHQ It. 3 < 2)	Women by Age (Fisher Exact Test) p	Women Sleep Disturbance Without Depressive Episode	Women by Age (Fisher Exact Test) p
**>75**	58 (8.0%)	3.4% (2/58)	Pivot	20.7% (12/58)	Pivot	92 (11.9%)	0% (0/92)	Pivot	18.5% (17/92)	Pivot
**60–74**	132 (18.1%)	1.5% (2/132)	0.587	9.8% (9/132)	0.010	150 (20.2%)	2.7% (4/150)	0.301	8% (12/150)	0.023
**45–59**	185 (25.4%)	1.1% (2/185)	0.242	6.5 (12/185)	0.004	190 (25.5%)	1.0% (2/190)	0.999	14.7% (18/190)	0.036
**30–44**	211 (30.0%)	2.8% (6/211)	0.683	4.7% (4/211)	0.0001	206 (27.7%)	4.3% (9/206)	0.061	5.8% (12/206)	0.001
**16–29**	142 (19.5%)	0.70 (1/142)	0.202	8.4% (12/142)	0.028	136 (17.6%)	2.2% (3/136)	0.275	8.0% (11/136)	0.024
Total		9/32 (28.1%—Prevalence 1.2)		49/70 (70%—Prevalence 6.7)			18/82 (21.9%—Prevalence 2.3)		70/144 (48.6%—Prevalence 9.0)	
Men vs Women		Chi sq- = 0.485 *p* = 0.486 OR = 1.4 (0.5–3.5)		Chi sq- = 8.729 * p = 0.003 OR = 2.5 (1.3–4.5)					Chi sq- = 2.752 ** p = 0.097 OR = 0.7 (0.5–1.1)	
High Discr.		>75 vs. others Fisher *p* = 0.153 **		60–74 vs. 30–44 Fisher *p* = 0.103			>75 vs. others Fisher *p* = 0.103 **		45–59 vs. 16–29 Fisher *p* = 0.699	

* Calculated as crude frequencies on depressive episodes, not as prevalence. ** Calculated as prevalence. *** PHQ = Patient Health Questionnaire.

## Data Availability

The data presented in this study are available upon request from the corresponding author. The data are not publicly available due to privacy and ethical issues.

## References

[1] Sedler M.J. (2016). Medicalization in Psychiatry: The Medical Model, Descriptive Diagnosis, and Lost Knowledge. Med. Health Care Philos..

[2] Huda A.S. (2021). The Medical Model and Its Application in Mental Health. Int. Rev. Psychiatry.

[3] Vandenbroucke J.P., von Elm E., Altman D.G., Gøtzsche P.C., Mulrow C.D., Pocock S.J., Poole C., Schlesselman J.J., Egger M., MD for the STROBE initiative (2007). Strengthening the Reporting of Observational Studies in Epidemiology (STROBE): Explanation and Elaboration. Ann. Intern. Med..

[4] Pearson M.L., Selby J.V., Katz K.A., Cantrell V., Braden C.R., Parise M.E., Paddock C.D., Lewin-Smith M.R., Kalasinsky V.F., Goldstein F.C. (2012). Clinical, Epidemiologic, Histopathologic and Molecular Features of an Unexplained Dermopathy. PLoS ONE.

[5] Parker G. (2008). How Should Mood Disorders Be Modelled?. Aust. N. Z. J. Psychiatry.

[6] Parker G. (2006). The DSM Classification of Depressive Disorders: Debating Its Utility. Can. J. Psychiatry.

[7] American Psychiatric Association (2013). Diagnostic and Statistical Manual of Mental Disorders: DSM-5.

[8] Angst J., Merikangas K.R., Cui L., Van Meter A., Ajdacic-Gross V., Rössler W. (2018). Bipolar Spectrum in Major Depressive Disorders. Eur. Arch. Psychiatry Clin. Neurosci..

[9] Benazzi F., Koukopoulos A., Akiskal H.S. (2004). Toward a Validation of a New Definition of Agitated Depression as a Bipolar Mixed State (Mixed Depression). Eur. Psychiatry.

[10] Akiskal H.S., Akiskal K.K. (2007). In Search of Aristotle: Temperament, Human Nature, Melancholia, Creativity and Eminence. J. Affect. Disord..

[11] Kalcev G., Cossu G., Preti A., Littera M.T., Frau S., Primavera D., Zaccheddu R., Matza V., Ermellino M., Pintus E. (2023). Development and Validation of the Questionnaire for Adaptive Hyperactivity and Goal Achievement (AHGA). Clin. Pract. Epidemiol. Ment. Health.

[12] Carta M.G., Kalcev G., Scano A., Primavera D., Orrù G., Gureye O., Cossu G., Nardi A.E. (2023). Is Bipolar Disorder the Consequence of a Genetic Weakness or Not Having Correctly Used a Potential Adaptive Condition?. Brain Sci..

[13] Kalcev G., Scano A., Orrù G., Primavera D., Cossu G., Nardi A.E., Carta M.G. (2023). Is a Genetic Variant Associated with Bipolar Disorder Frequent in People without Bipolar Disorder but with Characteristics of Hyperactivity and Novelty Seeking?. Clin. Pract. Epidemiol. Ment. Health.

[14] Primavera D., Aviles Gonzalez C.I., Romano F., Kalcev G., Pinna S., Minerba L., Scano A., Orrù G., Cossu G. (2024). Does the Response to a Stressful Condition in Older Adults with Life Rhythm Dysregulations Provide Evidence of the Existence of the “Dysregulation of Mood, Energy, and Social Rhythms Syndrome”?. Healthcare.

[15] Carta M.G., Kalcev G., Fornaro M., Pinna S., Gonzalez C.I.A., Nardi A.E., Primavera D. (2023). Does Screening for Bipolar Disorders Identify a “Dysregulation of Mood, Energy, and Social Rhythms Syndrome” (DYMERS)? A Heuristic Working Hypothesis. J. Clin. Med..

[16] Zelviene P., Kazlauskas E. (2018). Adjustment Disorder: Current Perspectives. Neuropsychiatr. Dis. Treat..

[17] Maercker A., Einsle F., Köllner V. (2007). Adjustment Disorders as Stress Response Syndromes: A New Diagnostic Concept and Its Exploration in a Medical Sample. Psychopathology.

[18] Strain J.J., Klipstein K., Newcorn J.H. (2008). Adjustment Disorders. Psychiatry.

[19] Patten S.B. (2009). Accumulation of Major Depressive Episodes over Time in a Prospective Study Indicates That Retrospectively Assessed Lifetime Prevalence Estimates Are Too Low. BMC Psychiatry.

[20] Newman S.C., Bland R.C. (1998). Incidence of Mental Disorders in Edmonton: Estimates of Rates and Methodological Issues. J. Psychiatr. Res..

[21] Carta M.G., Angst J. (2016). Screening for Bipolar Disorders: A Public Health Issue. J. Affect. Disord..

[22] Pan C., Ye J., Wen Y., Chu X., Jia Y., Cheng B., Cheng S., Liu L., Yang X., Liang C. (2022). The Associations between Sleep Behaviors, Lifestyle Factors, Genetic Risk and Mental Disorders: A Cohort Study of 402 290 UK Biobank Participants. Psychiatry Res..

[23] Solelhac G., Imler T., Strippoli M.-P.F., Marchi N.A., Berger M., Haba-Rubio J., Raffray T., Bayon V., Lombardi A.S., Ranjbar S. (2024). Sleep Disturbances and Incident Risk of Major Depressive Disorder in a Population-Based Cohort. Psychiatry Res..

[24] Cretu J.B., Culver J.L., Goffin K.C., Shah S., Ketter T.A. (2016). Sleep, Residual Mood Symptoms, and Time to Relapse in Recovered Patients with Bipolar Disorder. J. Affect. Disord..

[25] Baglioni C., Spiegelhalder K., Lombardo C., Riemann D. (2010). Sleep and Emotions: A Focus on Insomnia. Sleep Med. Rev..

[26] Mendlewicz J. (2009). Sleep Disturbances: Core Symptoms of Major Depressive Disorder Rather than Associated or Comorbid Disorders. World J. Biol. Psychiatry.

[27] Murphy M., Peterson M.J. (2015). Sleep Disturbances in Depression. Sleep Med. Clin..

[28] Sateia M.J. (2014). International Classification of Sleep Disorders-Third Edition. Chest.

[29] (2023). American Academy of Sleep Medicine International Classification of Sleep Disorders.

[30] American Psychiatric Association (2000). Diagnostic and Statistical Manual of Mental Disorders, Fourth Edition: DSM-IV-TR.

[31] Chung K.-F., Yeung W.-F., Ho F.Y.-Y., Yung K.-P., Yu Y.-M., Kwok C.-W. (2015). Cross-Cultural and Comparative Epidemiology of Insomnia: The Diagnostic and Statistical Manual (DSM), International Classification of Diseases (ICD) and International Classification of Sleep Disorders (ICSD). Sleep Med..

[32] Forbes E.E., Bertocci M.A., Gregory A.M., Ryan N.D., Axelson D.A., Birmaher B., Dahl R.E. (2008). Objective Sleep in Pediatric Anxiety Disorders and Major Depressive Disorder. J. Am. Acad. Child Adolesc. Psychiatry.

[33] Wu X., Zhang N., Chao J., Liu Y., Zhang B. (2024). Sex-Specific in the Association between Depressive Symptoms and Risk of Cognitive Impairment in Chinese Older Adults. Arch. Psychiatr. Nurs..

[34] Chang Y.-H., Yang C.-T., Hsieh S. (2023). Social Support Enhances the Mediating Effect of Psychological Resilience on the Relationship between Life Satisfaction and Depressive Symptom Severity. Sci. Rep..

[35] Grygiel P., Dolata R., Humenny G., Muszyński M. (2024). Depressive Symptoms and Loneliness among Early Adolescents: A Psychometric Network Analysis Approach. J. Child Psychol. Psychiatry.

[36] Wang J. (2024). The Longitudinal Relationship between Leisure Activities and Depressive Symptoms among Older Chinese Adults: An Autoregressive Cross-Lagged Analysis Approach. BMC Public Health.

[37] Rith-Najarian L.R., Boustani M.M., Chorpita B.F. (2019). A Systematic Review of Prevention Programs Targeting Depression, Anxiety, and Stress in University Students. J. Affect. Disord..

[38] Buonomo A., Benassi F., Gallo G., Salvati L., Strozza S. (2024). In-between Centers and Suburbs? Increasing Differentials in Recent Demographic Dynamics of Italian Metropolitan Cities. Genus.

[39] Moro M.F., Angermeyer M.C., Matschinger H., Holzinger A., Piras A.P., Cutrano F., Mura G., Carta M.G. (2015). Whom to Ask for Professional Help in Case of Major Depression? Help-Seeking Recommendations of the Sardinian Public. Adm. Policy Ment. Health.

[40] Perra A., Galetti A., Zaccheddu R., Locci A., Piludu F., Preti A., Primavera D., Di Natale L., Nardi A.E., Kurotshka P.K. (2023). A Recovery-Oriented Program for People with Bipolar Disorder through Virtual Reality-Based Cognitive Remediation: Results of a Feasibility Randomized Clinical Trial. J. Clin. Med..

[41] Kroenke K., Spitzer R.L., Williams J.B.W. (2001). The PHQ-9. J. Gen. Intern. Med..

[42] Rizzo R., Piccinelli M., Mazzi M.A., Bellantuono C., Tansella M. (2000). The Personal Health Questionnaire: A New Screening Instrument for Detection of ICD-10 Depressive Disorders in Primary Care. Psychol. Med..

[43] Mazzotti E., Fassone G., Picardi A., Sagoni E., Ramieri L., Lega I., Camaioni D., Abeni D., Pasquini P. (2003). Il Patient Health Questionnaire (PHQ) per lo screening dei disturbi psichiatrici: Uno studio di validazione nei confronti della Intervista Clinica Strutturata per il DSM-IV asse I (SCID-I). Off. J. Ital. Soc. Psychopathol..

[44] Pez O., Gilbert F., Bitfoi A., Carta M.G., Jordanova V., Garcia-Mahia C., Mateos-Alvarez R., Prince M., Tudorache B., Blatier C. (2010). Validity across Translations of Short Survey Psychiatric Diagnostic Instruments: CIDI-SF and CIS-R versus SCID-I/NP in Four European Countries. Soc. Psychiatry Psychiatr. Epidemiol..

[45] Smith D.J., Kyle S., Forty L., Cooper C., Walters J., Russell E., Caesar S., Farmer A., McGuffin P., Jones I. (2008). Differences in Depressive Symptom Profile between Males and Females. J. Affect. Disord..

[46] Serrano D., Martí-Lluch R., Cárdenas M., Solanas P., Marrugat J., Vilalta-Franch J., Garre-Olmo J. (2022). Gender Analysis of the Frequency and Course of Depressive Disorders and Relationship with Personality Traits in General Population: A Prospective Cohort Study. J. Affect. Disord..

[47] Barros M.B.d.A., Medina L.d.P.B., Lima M.G., de Azevedo R.C.S., Sousa N.F.d.S., Malta D.C. (2021). Association between Health Behaviors and Depression: Findings from the 2019 Brazilian National Health Survey. Rev. Bras. Epidemiol..

[48] Kibitov A.A., Rakitko A.S., Kasyanov E.D., Rukavishnikov G.V., Kozlova K.A., Ilinsky V.V., Neznanov N.G., Mazo G.E., Kibitov A.O. (2021). Screening of Depressive Symptoms in a Russian General Population Sample: A Web-Based Cross-Sectional Study. Clin. Pract. Epidemiol. Ment. Health.

[49] Horwath E., Cohen R.S., Weissman M.M. (2002). Epidemiology of Depressive and Anxiety Disorders. Textbook in Psychiatric Epidemiology.

[50] Huang L., Tang S., Rietkerk J., Appadurai V., Krebs M.D., Schork A.J., Werge T., Zuber V., Kendler K., Cai N. (2024). Polygenic Analyses Show Important Differences Between Major Depressive Disorder Symptoms Measured Using Various Instruments. Biol. Psychiatry.

[51] Kessler R.C., Andrews G., Mroczek D., Ustun B., Wittchen H.-U. (1998). The World Health Organization Composite International Diagnostic Interview Short-Form (CIDI-SF). Int. J. Methods Psychiatr. Res..

[52] Salvatore P., Indic P., Khalsa H.K., Tohen M., Baldessarini R.J., Maggini C. (2023). Circadian Activity Rhythms and Psychopathology in Major Depressive Episodes. Psychopathology.

[53] Roloff T., Haussleiter I., Meister K., Juckel G. (2022). Sleep Disturbances in the Context of Neurohormonal Dysregulation in Patients with Bipolar Disorder. Int. J. Bipolar Disord..

[54] Skeppar P., Adolfsson R. (2006). Bipolar II and the Bipolar Spectrum. Nord. J. Psychiatry.

[55] El-Mallakh R.S., Ghaemi S.N., Sagduyu K., Thase M.E., Wisniewski S.R., Nierenberg A.A., Zhang H.W., Pardo T.A., Sachs G. (2008). Antidepressant-Associated Chronic Irritable Dysphoria (ACID) in STEP-BD Patients. J. Affect. Disord..

[56] Primavera D., Cossu G., Marchegiani S., Preti A., Nardi A.E. (2024). Does the Dysregulation of Social Rhythms Syndrome (DYMERS) Be Considered an Essential Component of Panic Disorders?. Clin. Pract. Epidemiol. Ment. Health.

[57] Giovanni Carta M., Kalcev G., Scano A., Aviles Gonzalez C.I., Ouali U., Pinna S., Carrà G., Romano F., Preti A., Orrù G. (2023). The Impact of MDQ Positivity on Quality of Life Impairment: Does It Support the Hypothesis of “Dysregulation of Mood, Energy, and Social Rhythms Syndrome” (DYMERS)?. J. Public Health Res..

[58] Liu C., Ye Z., Chen L., Wang H., Wu B., Li D., Pan S., Qiu W., Ye H. (2024). Interaction Effects between Sleep-Related Disorders and Depression on Hypertension among Adults: A Cross-Sectional Study. BMC Psychiatry.

[59] Sullivan E.C., James E., Henderson L.-M., McCall C., Cairney S.A. (2023). The Influence of Emotion Regulation Strategies and Sleep Quality on Depression and Anxiety. Cortex.

[60] Fries G.R., Saldana V.A., Finnstein J., Rein T. (2023). Molecular Pathways of Major Depressive Disorder Converge on the Synapse. Mol. Psychiatry.

[61] Carta M.G., Ouali U., Perra A., Ben Cheikh Ahmed A., Boe L., Aissa A., Lorrai S., Cossu G., Aresti A., Preti A. (2021). Living With Bipolar Disorder in the Time of Covid-19: Biorhythms During the Severe Lockdown in Cagliari, Italy, and the Moderate Lockdown in Tunis, Tunisia. Front. Psychiatry.

[62] Alloy L.B., Walsh R.F.L., Smith L.T., Maddox M.A., Olino T.M., Zee P.C., Nusslock R. (2023). Circadian, Reward, and Emotion Systems in Teens Prospective Longitudinal Study: Protocol Overview of an Integrative Reward-Circadian Rhythm Model of First Onset of Bipolar Spectrum Disorder in Adolescence. BMC Psychiatry.

[63] Tassoni G., Cippitelli M., Scendoni R., Froldi R., Buratti E., Cerioni A., Mietti G., Cingolani M. (2024). A Study into the Nature and Extent of Polydrug Use in Driving Recidivism Behavior. Traffic INJ Prev..

[64] Pascale A., Negrin A., Laborde A. (2010). Cocaine base paste: Experience from the Montevideo Poison Control Center. Adicciones.

[65] Busch V., De Leeuw J.R.J. (2014). Unhealthy Behaviors in Adolescents: Multibehavioral Associations with Psychosocial Problems. Int.J. Behav. Med..

[66] Carta M., Preti A., Akiskal H. (2018). Coping with the New Era: Noise and Light Pollution, Hperactivity and Steroid Hormones. Towards an Evolutionary View of Bipolar Disorders. Clin. Pract. Epidemiol. Ment. Health.

[67] Smolensky M.H., Sackett-Lundeen L.L., Portaluppi F. (2015). Nocturnal Light Pollution and Underexposure to Daytime Sunlight: Complementary Mechanisms of Circadian Disruption and Related Diseases. Chronobiol. Int..

[68] Zhang C., Zhu Z., Zhao J., Li Y., Zhang Z., Zheng Y. (2023). Ubiquitous Light-Emitting Diodes: Potential Threats to Retinal Circadian Rhythms and Refractive Development. Sci. Total Environ..

[69] Oh J.H., Yoo H., Park H.K., Do Y.R. (2015). Analysis of Circadian Properties and Healthy Levels of Blue Light from Smartphones at Night. Sci. Rep..

[70] Martel J., Chang S.-H., Chevalier G., Ojcius D.M., Young J.D. (2023). Influence of Electromagnetic Fields on the Circadian Rhythm: Implications for Human Health and Disease. Biomed. J..

[71] Mortazavi S.A.R., Mortazavi S.M.J. (2018). Women with Hereditary Breast Cancer Predispositions Should Avoid Using Their Smartphones, Tablets and Laptops at Night. Iran. J. Basic Med. Sci..

[72] Skwarło-Sońta K., Jagota A. (2023). Achieving Healthy Aging in the Light-Polluted World. Sleep and Clocks in Aging and Longevity.

[73] Jeon B., Baek J. (2023). Menstrual Disturbances and Its Association with Sleep Disturbances: A Systematic Review. BMC Women’s Health.

[74] Lin Y., Hu Y., Guo J., Chen M., Xu X., Wen Y., Yang L., Lin S., Li H., Wu S. (2022). Association between Sleep and Multimorbidity in Chinese Elderly: Results from the Chinese Longitudinal Healthy Longevity Survey (CLHLS). Sleep Med..

[75] Feinsilver S.H., Hernandez A.B. (2017). Sleep in the Elderly: Unanswered Questions. Clin. Geriatr. Med..

[76] Kim J.H., Duffy J.F. (2018). Circadian Rhythm Sleep-Wake Disorders in Older Adults. Sleep Med. Clin..

[77] Feinsilver S.H. (2021). Normal and Abnormal Sleep in the Elderly. Clin. Geriatr. Med..

[78] Kocevska D., Lysen T.S., Dotinga A., Koopman-Verhoeff M.E., Luijk M.P.C.M., Antypa N., Biermasz N.R., Blokstra A., Brug J., Burk W.J. (2021). Sleep Characteristics across the Lifespan in 1.1 Million People from the Netherlands, United Kingdom and United States: A Systematic Review and Meta-Analysis. Nat. Hum. Behav..

[79] O’Connor E.A., Whitlock E.P., Beil T.L., Gaynes B.N. (2009). Screening for Depression in Adult Patients in Primary Care Settings: A Systematic Evidence Review. Ann. Intern. Med..

[80] El-Den S., Chen T.F., Gan Y.-L., Wong E., O’Reilly C.L. (2018). The Psychometric Properties of Depression Screening Tools in Primary Healthcare Settings: A Systematic Review. J. Affect. Disord..

[81] Katona C., Livingston G., Manela M., Leek C., Mullan E., Orrell M., D’Ath P., Zeitlin D. (1997). The Symptomatology of Depression in the Elderly. Int. Clin. Psychopharmacol..

[82] Kessler R.C., Birnbaum H., Bromet E., Hwang I., Sampson N., Shahly V. (2010). Age Differences in Major Depression: Results from the National Comorbidity Survey Replication (NCS-R). Psychol. Med..

